# Multi‐omic characterization of consensus molecular subtype 1 (CMS1) colorectal cancer with dampened immune response improves precision medicine

**DOI:** 10.1002/1878-0261.70023

**Published:** 2025-07-11

**Authors:** Livia Concetti, Manuel Scimeca, Julia Bischof, Jonathan Woodsmith, Massimiliano Agostini, Cristina Fiorani, Yufang Shi, Eleonora Candi, Gerry Melino, Alessandro Mauriello, Giuseppe S. Sica

**Affiliations:** ^1^ Department of Experimental Medicine, TOR University of Rome “Tor Vergata” Italy; ^2^ Indivumed GmbH, Falkenried Hamburg Germany; ^3^ Department of Surgical Science University of Rome “Tor Vergata” Italy; ^4^ The Fourth Affiliated Hospital of Soochow University, Institutes for Translational Medicine, Suzhou Medical College Soochow University Suzhou China

**Keywords:** CMS1, immune response, colorectal cancer, JAK/STAT, personalized medicine

## Abstract

Colorectal cancer (CRC) is a heterogenous disease with distinct biological and clinical subgroups, each with different prognoses and responses to therapy. In this case report, taking inspiration from a case of locally advanced CRC with serine/threonine‐protein kinase B‐raf (BRAF) V600E mutation, we highlight an atypical consensus molecular subtype 1 (CMS1). Deep multi‐omic analyses showed a limited expression of programmed cell death protein 1 (PD‐1) and reduced T‐cell infiltration, including CD8^+^ and natural killer (NK) cells, in the analyzed CMS1 tumor. In parallel, a reduced activation of the JAK/STAT pathway was detected, suggesting a lack of clinical response to immunotherapy with checkpoint inhibitors. Furthermore, the finding of up‐regulated expression of WEE1 G2 checkpoint kinase (WEE1), checkpoint kinase 1 (CHK1), and checkpoint kinase 2 (CHK2), poly(ADP‐ribose) polymerase (*PARP*), and heat shock protein 90 (*HSP90*) suggests a potential alternative therapeutic approach using inhibitors of the cell cycle, HSP90, or PARP in combination with conventional chemotherapy, targeted agents, or immunotherapy. This paradigmatic case should stimulate a regular deep omics analysis to improve precision medicine. We therefore suggest that full mutational and expression profiling analyses of CRC subtypes should be undertaken to improve therapeutic strategies in CRC treatment.

## Graphical abstract

This study highlights the importance of multi‐omic analyses in characterizing colorectal cancers. Indeed, our analysis revealed a rare CMS1 exhibiting dampened immune activation, including reduced PD‐1 expression, moderate CD8^+^ T‐cell infiltration, and suppressed JAK/STAT pathway. These findings suggest a reduced response to immune checkpoint inhibitors and propose alternative therapeutical strategies focused on cell cycle regulators such as HSP90, and PARP.

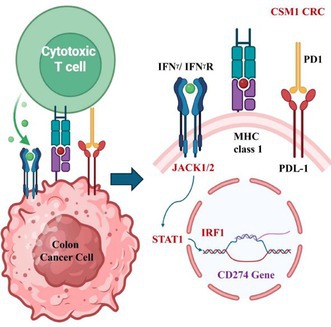

**Abbreviations**
ALK: anaplastic lymphoma kinase
BRAF: serine/threonine‐protein kinase B‐raf
CHK1: checkpoint kinase 1
CHK1/2: checkpoint kinase 1/2
CHK2: checkpoint kinase 2
CIMP: CpG island methylation phenotype
CMS1: consensus molecular subtype 1
CRC: colorectal cancer
dMMR: mismatch repair‐deficient
FFPE: formalin‐fixed and paraffin‐embedded
FGFR2: fibroblast growth factor receptor 2
GATK: Genome Analysis Toolkit
GOPC: coiled‐coil motif‐containing protein
H&E: Hematoxylin and Eosin
HSP90: heat shock protein 90
HSP90AA1: heat shock protein 90 alpha family class A member 1
ICI: immune checkpoint inhibitors
MLH1: mutL Homolog 1
MSH: mutS Homolog
MSI: microsatellite instability
NGS: next‐generation sequencing
NK: natural killer
NTRK1: neurotrophic tyrosine kinase receptor
PARP: poly(ADP‐ribose) polymerase
PD‐1: programmed cell death protein 1
PD‐L1: programmed cell death‐ligand 1
PMS2: mismatch repair system component
ROS1: receptor tyrosine kinase
Th1: T helper cells
TMB: tumoral mutational burden
TME: tumor microenvironment
TPM: transcripts per million
WEE1: WEE1 G2 checkpoint kinase
WGS: whole genome sequencing

## 1 Introduction

Colorectal cancer (CRC) is the second leading cause of cancer‐related deaths worldwide. Large geographical variations in incidence and mortality rates are observed; overall, the lifetime risk of developing CRC is about 1 in 23 for men and 1 in 26 for women [1]. In 2020, more than 930 000 deaths due to CRC were estimated to have occurred worldwide [2, 3]. The vast majority of CRC can be classified as sporadic (70–75%) or eventually familial (20–25%); only 5% are hereditary. CRC is thought to be caused by genetic alterations targeting tumor suppressor genes, oncogenes, and DNA‐repair genes [4, 5, 6, 7, 8, 9]. The progression of adenoma to carcinoma is a multistep process, and genomic instability seems to play a central role that leads to the accretion of other potential genetic aberrations responsible for the transformation [10, 11, 12]. Chromosomal instability (CIN) is the most common (85% of total CRC) genetic mechanism occurring in CC, and CpG island methylation phenotype (CIMP) is predominantly observed in the proximal colon [13].

This heterogeneity makes CRC a clinically diverse disease and makes it difficult to determine which patients will benefit most from adjuvant therapy and complicates the development of new targeted agents. According to the molecular and biological profile, four main molecularly distinct subtypes can be recognized, namely consensus molecular subtypes (CMS) 1 to 4 [14, 15, 16]. CMS1 represents only 14% of total CRC; The CMS2 subtype is more common (34%) and corresponds to the canonical subtype with high CIN; CMS3 corresponds to the metabolic subtype (10% of CRC), and the CMS4 corresponds to the mesenchymal subtype (25% of all cases). CMS1 (microsatellite instability (MSI) immune subtype) is characterized by hypermutation and enrichment for BRAFV600E and is associated with hypermethylation of the CpG island (CIMP^+^ phenotype), which causes loss of tumor suppressor function. Frequent in CMS1 tumors, immune cells such as T‐lymphocytes and natural killer lymphocytes can be found in the tumor microenvironment, displaying a strong immune activation [17]. Indeed, JAK/STAT and MAPK pathways are typically activated in CMS1 [13]. Precursors of these tumors are frequently serrated lesions of the right colon. Prognostic outcome is better when compared to patients with microsatellite stability, especially for early‐stage tumors [18, 19], but poor after relapse [20].

Treatment for colon cancer is based largely on the stage (0 to 4) of the cancer, but other factors can also be important. People with colon cancer that has not spread to distant sites usually have surgery as the main or first treatment [21, 22]. Chemotherapy may also be used after surgery or, whenever the tumor is metastatic or locally advanced, chemo is the main treatment and is usually given preoperatively or as the sole treatment [23, 24, 25]. Most people with stage IV cancer will get chemo and/or targeted therapies to control the cancer. Targeted drugs work differently from chemotherapy, and they sometimes work when chemo drugs do not [26]. They can be used either along with chemotherapy or by themselves if chemo is no longer working. Several types of targeted drugs might be used to treat CRC, and this pretty much depends on the molecular and biological tumor profile [27].

The fact that CMS1 cancers display a large immune infiltrate, including CD8^+^ cytotoxic T lymphocytes, CD4^+^ activated Type 1 T helper cells (Th1), and natural killer cells, makes these tumors potentially susceptible to monoclonal antibody targeting of PD‐1/Programmed cell death‐ligand 1 (PD‐L1) [28, 29, 30, 31]; however, it is important to remember that the anti‐PD‐L1 therapies in CRC are currently chosen on the basis of mismatch repair‐deficient(dMMR)/MSI‐H status rather than CMS subtyping.

Only about one‐third of patients respond to immunotherapy, presenting a significant challenge that highlights the need for a more accurate assessment of the immune status of tumors, including the CMS1 profile. Additionally, the tumor stroma, or more broadly the tumor microenvironment (TME), is increasingly recognized as a critical factor influencing tumor immunotype and shaping the immune progression and outcome of cancer cells. According to this, cancers can be categorized into distinct immunological phenotypes: immune‐inflamed (characterized by a high presence of immune cells within the tumor), immune‐excluded (where T cells are confined to the stroma and unable to penetrate the tumor), and immune‐desert (lacking any notable immune cell infiltration) [32]. Even with advancements in immune checkpoint inhibitors (ICIs), overcoming the challenges posed by immune‐excluded and immune‐desert phenotypes remains difficult. It is estimated that 60–70% of cancer patients exhibit immune‐restrictive phenotypes, which include most cases resistant to ICI therapies. Despite these challenges, ICIs, particularly those targeting the PD‐L1/PD‐1 pathway, have demonstrated outstanding success across different cancers. These therapies have expanded into perioperative settings, helping to lower relapse rates and providing long‐lasting clinical benefits. Moreover, combining chemotherapy with PD‐L1 inhibitors has shown promise in addressing immune resistance in the TME, although the results have been variable across different tumor types [33, 34].

This study aims to perform a complete deep omics molecular investigation of a CRC case in order to reveal specific alterations, which could help elucidating the underlying mechanisms of immune response and susceptibility to PD‐1/PD‐L1 blockade. This case report shows the power of deep multi‐omics opening new perspectives for targeted therapies; as such, it should foster regular multi‐omics investigations in all CRC patients.

## 2 Materials and methods

### 2.1 Collection of samples

All the procedures were carried out in compliance with the ethical standards of the institutional and/or national ethics committee and with the Helsinki Declaration of 1964 and its subsequent changes or with comparable ethics standards. Informed written voluntary consent was obtained from every participant in the study. The ethical committee of the Policlinico Tor Vergata approved the protocol in September 2019 (reference number #96‐19).

Tumor tissue was collected in March 2021 at the Policlinico Tor Vergata following a standardized protocol designed to minimize cold ischemia before freezing samples in liquid nitrogen [35, 36, 37, 38]. Serial sections stained with Hematoxylin and Eosin (H&E) Policlinico Tor Vergata were used for pathological quality control.

The inclusion criteria for tumor sample collection required a tumor content of at least 30%, necrosis of no more than 30%, and the presence of invasive tumor cells. Additionally, normal tissues were collected for comparison. Protein lysates and nucleic acids were extracted using 10 mg of each collected tissue sample, ensuring that the tissues remained frozen throughout the entire procedure.

For histological and immunohistochemical analysis, serial sections were prepared from formalin‐fixed and paraffin‐embedded (FFPE) blocks. Histological assessments were performed independently by two pathologists on H&E‐stained slides. Immunohistochemistry was used to evaluate the expression of key prognostic and predictive biomarkers in CRC, such as CDX2, CK7, and Chromogranin A [39]. These analyses were conducted using the automated Leica Bond IHC platform (Leica Biosystems, Deer Park, IL, USA). The primary antibodies employed included mouse monoclonal anti‐CK7 (clone RN7; Leica Biosystems) and mouse monoclonal anti‐Chromogranin A (clone 5H7; Leica Biosystems).

### 2.2 Nucleic acid extraction and quality assessment

As previously described, frozen tissue slices were used for nucleic acid extraction and quality assessment [40].

### 2.3 Library preparation and NGS sequencing

Libraries for whole genome sequencing (WGS) and whole transcriptome sequencing were performed as described by Yang et al. [40].

### 2.4 NGS data processing

To align next‐generation sequencing (NGS) data, the Grch38 genome assembly was used as a reference. As for the normal samples, the Haplotype Caller from the Genome Analysis Toolkit (GATK) was used for both identification and annotation of short genomic variations [41]. WGS somatic variations were called using a consensus of Mutect2 [42], strelka [43], varscan [44], and somatic sniper [45]. Structural variations were called using r packages titancna [46], DellyCNV and DellyCall [47], as well as manta [48].

RNA‐Seq differential expression was based on normalized read count data (TPM: transcripts per million).

### 2.5 Mass spectrometry data collection

For whole proteome profiling, 5–10 mg of fresh‐frozen tissue was used for whole proteome profiling as described by Han et al. [49].

Proteomics data were acquired by the DIA method, consisting of one full range MS1 scan and 50 DIA segments, which was adapted from Bruderer et al. [50].

The Tissue‐specific spectral libraries were generated by combining high‐fractionated DDA and DIA measurements on a pool of tissue material and raw data processed using biognosys' software spectronaut 13.

### 2.6 Bioinformatical analyses

Mutational signatures were calculated using the r package MutationalPatterns [51, 52, 53, 54]. MSI classification was done using the r package MSIseq [55]. Metrics to define chromosomal instability were determined using the r package CINmetrics [56] and CNHplus [57]. Aneuploidy events were analyzed using ascets [58]. Aneuploidy events span more than 90% of the chromosome. Visualization of results was done in igv [59]. Consensus molecular subtyping is based on RNA‐Seq data, using the r package cmscaller [60].

TMB was calculated as the number of non‐synonymous mutations of protein‐coding genes divided by exome size in Megabases.

## 3 Results and Discussion

**Fig. 1:**
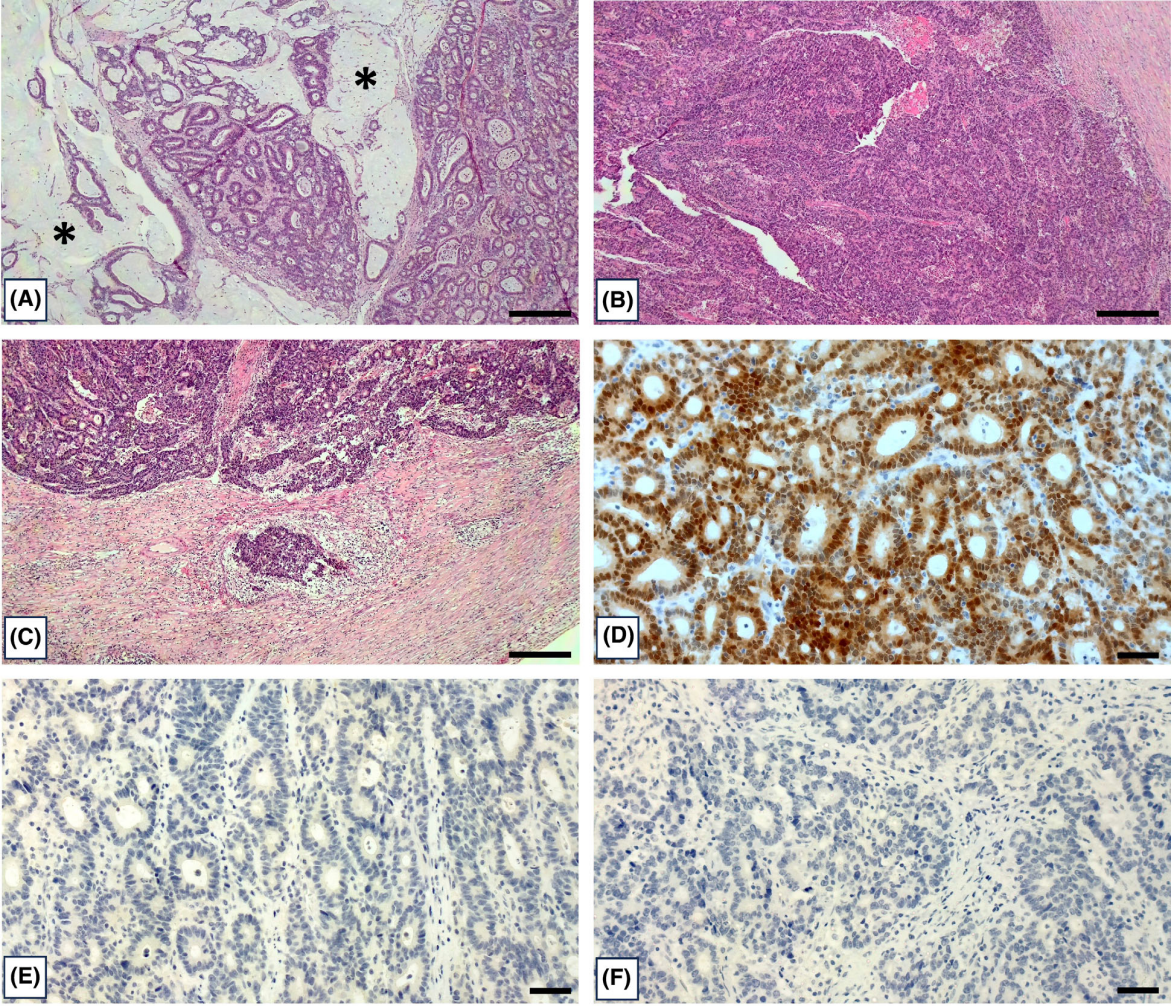
Histological and immunohistochemical analysis. (A) Hematoxylin and eosin staining shows a high‐grade colon adenocarcinoma with extensive mucinous features (The asterisks highlight the mucinous areas) (~ 30%). (B) Area of solid growth. (C) Image shows the area of tumor budding. (D) Immunohistochemical analysis displays numerous CDX2 positive colon cancer cells. (E) CK7 negative colon adenocarcinoma. (F) No expression of Chromogranin A. Scale bars represent 300 μm (panels A–C) or 50 μm (panels D, E).

Tissue samples were collected from the surgical specimen of an 82‐year‐old woman who underwent radical right colectomy with D3 lymphadenectomy and complete mesocolon excision for a cT4N0M0 bleeding tumor of the right colon [61]. Morphological analysis revealed a high‐grade adenocarcinoma with extensive mucinous features (~ 30%) (Fig. 1A) and full‐thickness infiltration of the bowel wall (Fig. 1B) (stage IIB, grade G3). Histologically, the lesion appeared ulcerated, with focal areas of solid growth (Fig. 1B) and extensive intra‐tumoral necrosis. The neoplasm exhibited an infiltrative growth pattern at the tumor's advancing front (Fig. 1C). Angioinvasion was not observed, but the neoplasm appeared highly vascularized. There was no perineural infiltration. No metastasis was observed in the 28 analyzed lymph nodes (TNM pT4a N0). Immunohistochemical investigations revealed positivity for CDX2 (Fig. 1D), whereas no expression of either Chromogranin A (Fig. 1E) or CK7 (Fig. 1F) was observed.

**Fig. 2:**
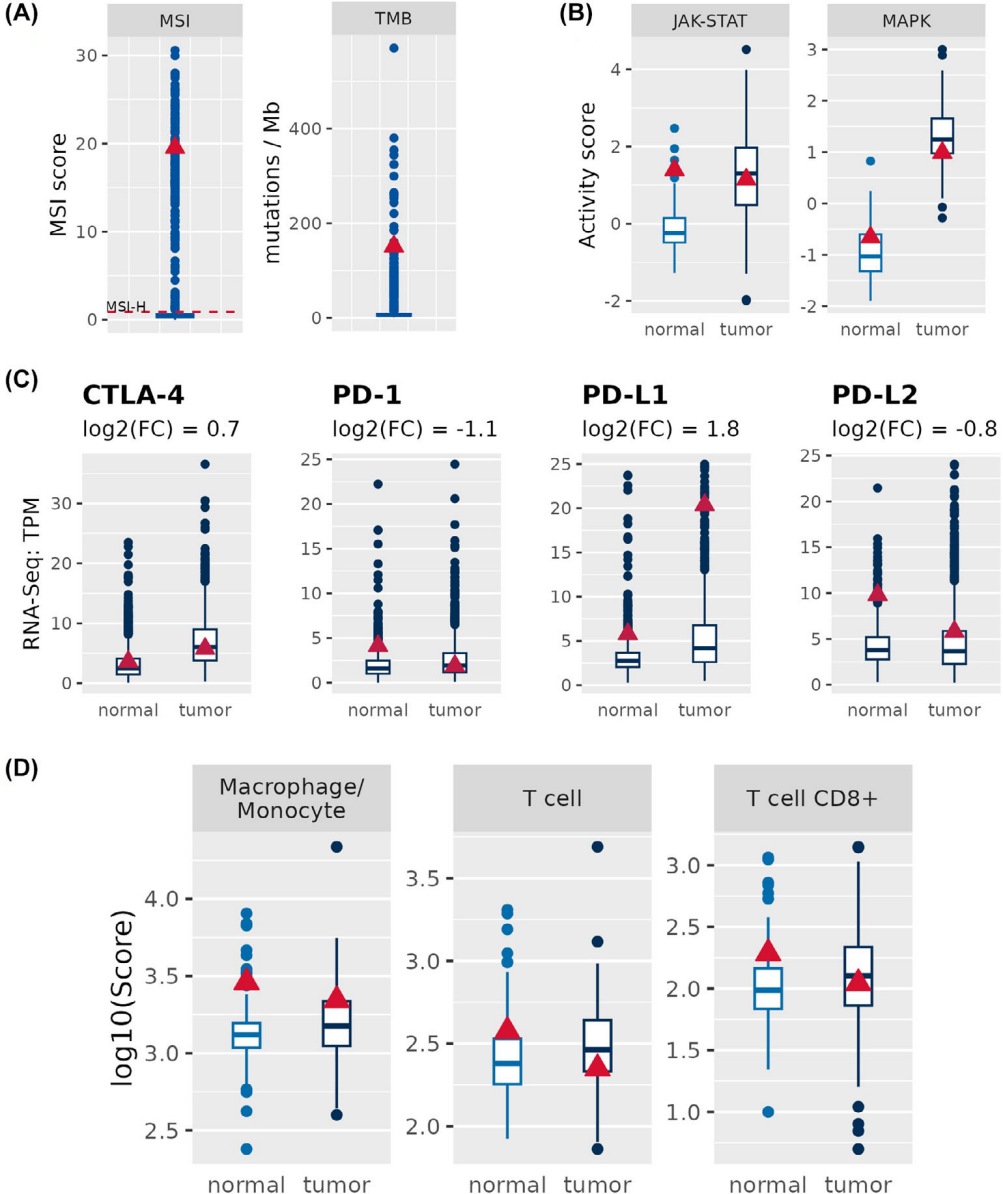
Mutational analysis, JAK/STAT pathway and expression levels of immune checkpoint in a Colorectal cancer (CRC) patient. (A) The patient shows higher Tumoral Mutational Burden (TMB) and Microsatellite Instability (MSI) when compared to the cohort of CRC patients. TMB patient = 151. The red broken line represents the threshold value beyond which the patient is classified as high MSI. (B) JAK–STAT signaling pathway is not activated in the CRC patient of interest. As aspect the MAPK is activated in this CRC patient. Boxplots indicate the values of CRC cohort (in B and C filtered for CMS1 patients only), and the red triangle refers to our CRC patient of interest. Graphs show mean, max and min values. (C) mRNA expression of PD‐1 = Programmed cell death protein‐1; PD‐L1 and PD‐L2 = Programmed death‐ligand 1 and 2; CTLA‐4 = Cytotoxic T‐lymphocyte antigen 4. PD‐L1 is highly expressed in tumor tissue compared to normal tissue. Whereas PD‐1 is down‐regulated and no differences between tumor and normal could be found for CTLA‐4 and PD‐L2. Boxplots indicate the values of CRC cohort, and the red triangle refers to our CRC patient of interest. (D) Macrophages and CD8^+^ T cells are both lower in tumor tissue compared to normal tissue of the CRC patient of interest.

For research purposes, in addition to regular pathology evaluation, the removed tumor was subjected to WGS, transcriptomic, and proteomic analysis. As reported in Table 1, these data were compared to our control cohort. This cohort included 1385 CRCs (age at surgery ranged from 24 to 97), classified from a molecular point of view as follows: 206 CMS1, 344 CMS2, 157 CMS3, and 420 CMS4. Based on transcriptome profiling, the patient was defined as CMS1. Right CRC is characterized by high MSI and, according to CMS, is classified as CMS1, which displays an increased expression of genes associated with immune infiltration, mainly composed of Th1 and CD8 T cells, along with strong activation of immune evasion pathways [17]. Indeed, the patient was classified as MSI‐H and high TMB (Fig. 2A). Moreover, this subtype is also associated with specific biological programs and distinct activated signaling pathways, particularly the activation of Janus kinase/signal transducers and activators of transcription (JAK–STAT). Several observations suggest that JAK/STAT inhibition might reduce the efficacy of immunotherapy [62, 63]. However, the isolated CMS1 tumor showed not only a reduced activation of the JAK/STAT pathway (Fig. 2B and Fig. S1) but also a reduction in both the expression levels of PD‐1 (Fig. 2C) and the T‐cell infiltration (Fig. 2D). Furthermore, some of the JAK/STAT components carry somatic mutations, particularly JAK2, JAK3 (JAK2: Arg1113His (VAF: 24.7%); JAK3: Glu1041Gly (VAF: 39.3%)) and STAT3 (Pro1217His (VAF: 28.8%)), as well as some MHC class I genes, including HLA‐B (Pro 209 frameshift (VAF: 32.6%)) and HLA‐C (Gly 50 Asp (VAF: 32.9%)). This might lead to a reduction in the expression levels of PD‐1 (Fig. 2C) Both inactivation of the JAK/STAT pathway and reduction of T cells can impact the expression of programmed death 1 (PD‐1). Indeed, a down‐regulation in the expression of PD‐1 was detected in the patient (Fig. 2C). In line with molecular investigations, immunohistochemical analysis showed high positivity for PD‐L1 by CRC cells but no/rare expression of CTLA4 and PD1 (see Fig. S2). For PD‐L1, a missense mutation was detected (Fig. S3), which, however, may not impact the expression (Fig. 2C). Although PD‐L1 is highly expressed, multiple other important components of the immune response (PD‐1/T cell/JAK/STAT) are altered in the tumor tissue and may reduce the immune response and thus dampen the response to immunotherapy. The combination of molecular characteristics reported here makes this CRC highly paradigmatic. In fact, the analyzed sample is characterized by several remarkable molecular features such as high microsatellite instability (MSI‐H), high TMB, PD1 fold change less than one 1 in down‐regulation, no change in CTLA‐4, reduction in JAK/STAT signaling, and high MAPK pathway activity (Fig. S2). In our control cohort, which includes 206 CMS1 cases, such a molecular profile is very rare, occurring in only seven cases (3.3%). The paradigmatic molecular features highlight the complexity and heterogeneity of CRC, demonstrating how detailed molecular profiling can provide critical insights for the development of patient‐tailored therapies. CIN in the patient was stable compared to other CRC patients; in fact, CMS1 is known to be stable at the level of CIN (Fig. S4A). Considering aneuploidy, we could identify chromosomal amplifications on chromosome arms 1q, 8p, 8q, 9p, and 9q (events are called if they span more than 90% of the chromosome); amplification on 8q and deletions on 8p have already been reported in CRC (Fig. S4B) [64, 65]. Also, we found genes FH, AKT3, and SMYD3 to be amplified; all of them are located on chromosome arm 1q. In addition to the previously reported mutation of BRAF (Val600Glu), which is characteristic of right colon cancer [17], using a comprehensive genomic mutational analysis, several somatic mutations were detected (Fig. S3). These mutations are currently being investigated as potential targeted treatments in several clinical trials. In detail, it was possible to isolate mutations affecting several receptor tyrosine kinases, including anaplastic lymphoma kinase (ALK), receptor tyrosine kinase (ROS1) [66], neurotrophic tyrosine kinase receptor (NTRK1) and Abelson (ABL) tyrosine kinase 1, which plays a key role in the pathogenesis of tumors including CC [67]. Of interest, the patient carried a mutation in the *ALK* gene (Gly1518Ser) with a substantially high variant allele frequency (VAF: 34.3%). This mutation is located on the intracellular domain of the protein, but outside the kinase domain (Fig. S3). This is, to our knowledge, a mutation that has not been previously described in CRC.

**Table 1:** Demographics of Indivumeds Colorectal cancer (CRC) cases used as background cohort (total: 1385). (A) Gender, age and tumor stage distribution. (B) MSI classification in CMS groups.

(A)		
Clinical parameter		# Cases
Gender	Female	582
	Male	803
Age at surgery	Min	24
	25% percentile	61
	50% percentile	70
	75% percentile	77
	Max	97
Tumor stage	I	168
	II	395
	III	339
	IV	463
	Unknown	20

(B)		
CMS classification		
CMS	MSI‐H	MSS
CMS1	119	87
CMS2	10	334
CMS3	16	141
CMS4	33	387

Another mutation detected in the present tumor was found in the Abelson tyrosine kinase 1 (ABL1) gene (Gly537Ser). This mutation lies outside of the kinase domain, within the conserved PXXP motifs that mediate binding to SH3 domain‐containing proteins (Fig. S3) [68]. Whether this mutation affects the interaction ability with its binding partners remains to be investigated. The ABL tyrosine kinases family composed of ABL1 and ABL2 plays a key role in promoting tumor progression and metastasis formation [69]. Although ABL1 somatic mutations in solid tumors are rare [70], in non‐small cell lung cancer, ABL1 mutations confer sensitivity to imatinib and dasatinib [71], suggesting that somatically mutated ABL1 may represent a novel druggable driver also in CRC.

A further mutation (Ala1416Thr) with a VAF of 32.2% of c‐Ros oncogene 1, receptor tyrosine kinase (ROS1) was observed in this patient. This mutation is located in a region of the protein that does not contain known functional domains (Fig. S3). However, it should be noted that in CRC the main ROS1 genomic alteration previously described is a fusion such as Golgi‐Associated PDZ And Coiled‐Coil Motif‐Containing Protein (*GOPC*)‐*ROS1* [66], Solute Carrier Family 34 Member 2 (*SLC34A2*)‐*ROS1* [72] and Tetratricopeptide Repeat Protein 28 (*TTC28*)‐*ROS1* [66, 67]. Even though, in non‐small cell lung cancer patients with ROS1 rearrangement are treated with crizotinib, a tyrosine kinase inhibitor [72], clinical trials on targeting ROS1 in CRC are very limited [73].

Besides mutations in receptor tyrosine kinases, a mutation (Tyr734Cys, VAF = 26.9%) in the fibroblast growth factor receptor 2 (FGFR2) was also observed, which is located in the intracellular kinase domain (Fig. S3). The fibroblast growth factor receptor family is highly expressed in CRC, and their expression correlates with tumor growth and invasiveness, as well as poor prognosis [74], indicating that a member of this family, in particular FGFR2, may represent a therapeutic molecular target [74]. However, no up‐regulation in FGFR2 expression was detected in our sample (Fig. S5).

**Fig. 3:**
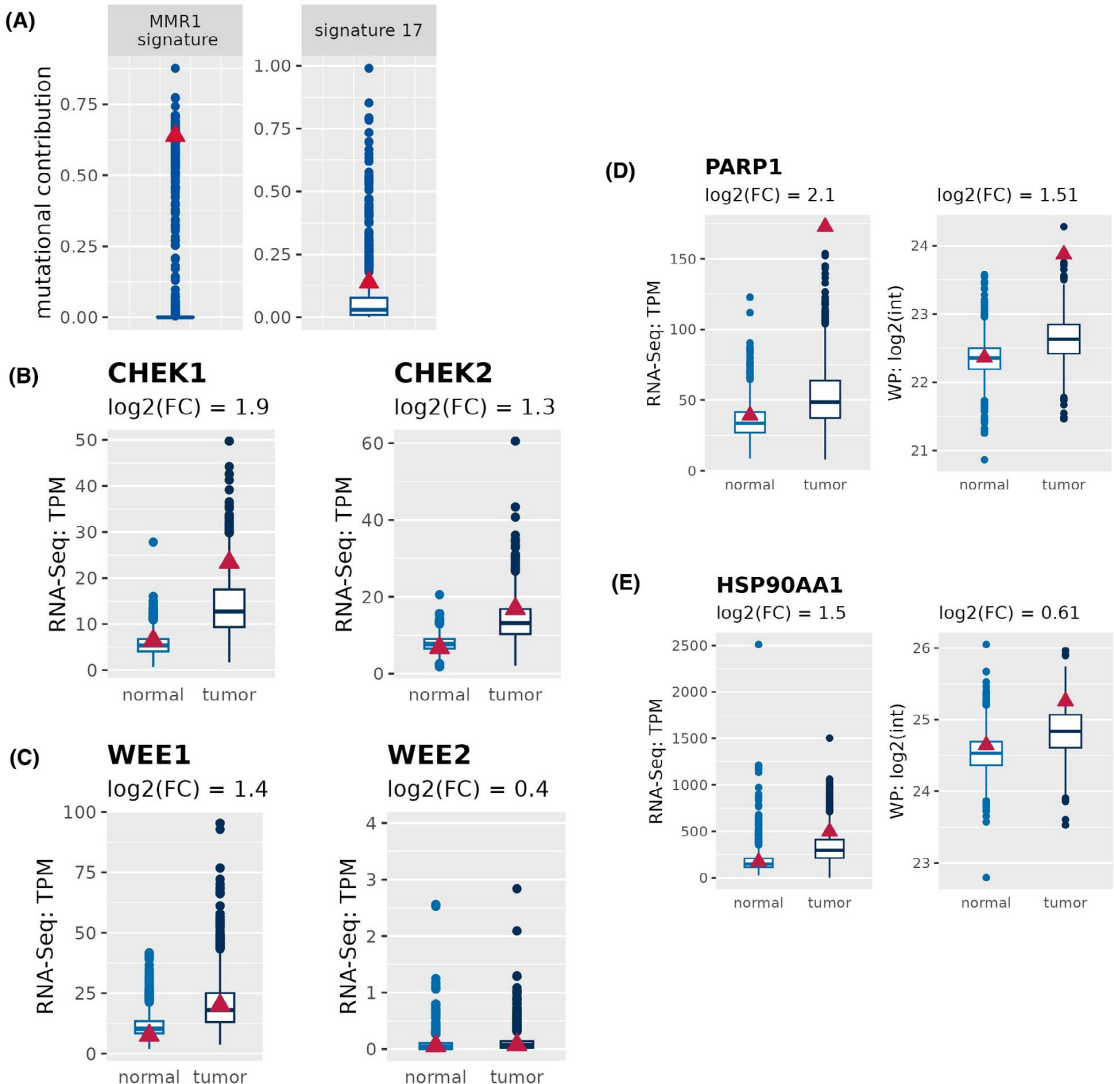
Mutational signature and expression of Poly(ADP‐Ribose) Polymerase 1 (PARP1) and Heat Shock Protein 90 Alpha family class A member 1 (HSP90AA1) in a Colorectal Cancer (CRC) patient. (A) The CRC patient displays a higher contribution in MMR1 and in Reference Signature 17 mutational signature (Ref. Sig. 17). (B) CHEK1 and CHEK2 are highly expressed in tumor tissue compared to normal tissue assessed by RNA‐Seq analysis. No differences were found for CHEK2 protein expression. (C) RNA‐seq analysis show high levels of WEE1 in tumor tissue when compared to normal tissue. CHEK1 = Checkpoint kinase 1; CHEK2 = Checkpoint kinase 2; WEE1 = WEE1 G2 Checkpoint Kinase; WEE2 = WEE2 Oocyte Meiosis Inhibiting Kinase. (D) PARP1 and (E) HSP90AA1 are highly expressed in tumor tissue compared to normal tissue both at RNA and protein levels. Boxplots indicate the values of CRC cohort patients, and the red triangle refers to our CRC patient of interest. Graphs show mean, max and min values.

The whole cancer genome sequencing of the patient's tumor tissue also displays an enriched contribution to MMR1 and Ref Sig B7 mutational signature (Fig. 3A). Ref Sig MMR1 is MMR deficiency‐associated SNV signatures which occur in cancers with inactivation of MutS Homolog (MSH) 2, MSH6, PMS1 Homolog 2, Mismatch Repair System Component (PMS2), or MutL Homolog 1 (MLH1) and is in particular associated with driver mutation in Ribosomal Protein L22 (RPL22). Moreover, Ref Sig 17 is primarily detected in esophagus and stomach cancers and is associated with high mutation burden, elevated neoantigen levels, and poor survival [75]. However, immunohistochemical data showed that MLH1and MSH2 were down‐regulated in the CRC sample, whereas both PMS2 and MSH6 were up‐regulated (Fig. S6). This datum indicates that both heterodimers (MLH1/PMS2 or MSH2/MSH6) are involved in the MSI status. This evidence further underlines the peculiar molecular characteristics of the analyzed CRC. In fact, generally only one heterodimer is affected in dMMR/MSI CRCs. A recent study of Reitsam et al. [76] reported the involvement of both heterodimers in about 1% of analyzed digestive cancers. The RNA‐Seq data are consistent for MLH1, PMS2, and MSH6, but reveal a discrepancy for MSH2. In fact, while immunohistochemical analysis indicates an impairment in MSH2, the molecular data show increased expression levels compared to the control cohort (Fig. S6). This discrepancy may be due to post‐transcriptional modifications that prevent mRNA translation into functional protein, thus resulting in lower detectable protein. Clinical studies revealed that patients with high contributions in Ref Sig 17 are more sensitive to inhibitors of the cell cycle checkpoint regulators WEE1 G2 Checkpoint Kinase (WEE1) and Checkpoint Kinase 1/2 (CHK1/2). Of note, an up‐regulation of proteins involved in cell cycle checkpoints such as CHK1/2 (Fig. 3B) and WEE1 were detected (Fig. 3C). Moreover, this patient has a somatic mutation in CTNNB1 (Asp 32 Asn; VAF: 19%), EXO1 (Ile 123 Thr; VAF: 4%), MLH3 (Arg 381 Cys; VAF: 5.4%), MSH6 (Arg 361 His; VAF: 29.4%) and PMS1 (Lys 163 frameshift; VAF: 47.8%).

The finding of an up‐regulation both at mRNA and protein levels of PARP1 and HSP90AA1, together with a BRCA2 mutation, suggests a potential alternative therapy with PARP and HSP90 inhibitors, respectively (Fig. 3B,D). The use of PARP inhibitors is further supported by the deficiency in the homologous recombination (HR) repair mechanism observed in our patient case, which is mainly due to the somatic and germline mutations affecting genes involved in HR, including TOP3A and TP53BP. Indeed, tumors with HR deficiency are more sensitive to PARP inhibitors [77]. Cell cycle inhibitors (WEE1 and CHK1/2 up‐regulated) or (HSP90) inhibitors (HSP90AA1 up‐regulated) in combination with chemotherapy may be a valid alternative therapeutic approach.

## 4 Conclusion

In the era of personalized medicine, the identification of new biomarkers associated with biological events involved in both cancer occurrence and progression, such as cell death [78, 79, 80, 81, 82, 83, 84], cancer‐associated inflammation [85], tumor invasion, and metastasis [86, 87, 88, 89, 90], lays the foundation for the identification of innovative patient‐tailored therapies. Among them, immunotherapy has long been explored as a possible treatment for patients affected by CMS1 CRC showing a favorable progression‐free survival [91]. However, the response to immunomodulatory therapies is a complex system that involves the interactions of numerous molecular pathways [32]. In this context, present findings showed that not all CMS1 MSI immune tumors would respond to standard treatments [92] due to the dysregulation of pathways involved in the immune checkpoints expression, such as JAK/STAT signaling. By unraveling the molecular determinants underlying the observed low evidence for immunotherapy, this report aims to pave the way for more targeted and efficacious therapeutic approaches tailored to the specific needs of CMS colorectal cancer patients.

## Conflict of interest

The authors declare no conflict of interest.

## Author contributions

GM, JW, JB, EC, AM, MS, YS, and GSS conceived the project; LC, JW, JB, GM, CF, and MA wrote the manuscript. LC, JW, JB, MS, and MA prepared figs. LC, CF, MS, and MA performed the analysis. GM, MA, JB, and GSS interpreted the data. All of the authors have approved this submitted version.

## Supporting information


**Fig. S1.** Image shows RNA‐Seq analysis of the JAK/STAT pathway (JAK2, JAK3, and STAT3) in tumor tissue when compared to normal tissue. Boxplots indicate the values of CRC cohort patients, and the red triangle refers to our CRC patient of interest. Graphs show mean, max, and min values.
**Fig. S2.** Immunohistochemical analysis of immune checkpoints and Consensus molecular subtype (CMS) 1 classification. (A) Programmed death‐ligand 1 (PDL‐1) expression in the analyzed colon adenocarcinoma. (B) High‐magnification of panel A shows numerous PDL‐1 positive colon cancer cells. (C) No/moderate expression of Cytotoxic T‐lymphocyte antigen 4 (CTLA4) in colon cancer cells. (D) No expression of Programmed cell death 1 (PD‐1). (E) Classification of CMS1 cases in terms of Microsatellite Instability (MSI) status (blue: MSI‐H), Microsatellite Instability (TMB) (blue: TMB > 75% percentile of all CRC cases), CTLA‐4 and PD‐1 expression (blue: low: tumor/normal log2FC < 1), as well as JAK–STAT and MAPK activity (blue: JAK–STAT tumor/normal differences < 0, MAPK difference > 0). Scale bars represent 50 μm panels A, C, D and 40 μm panel B.
**Fig. S3.** Mutational analysis. (A) The most relevant somatic mutation detected in our colorectal cancer (CRC) patient, along with the currently active clinical trials considering this mutation for targeted therapies. (B–E) Schematic representation of the indicated genes and location of frequent pathogenic mutations described in CRC patients (TCGA, PanCancer Atlas) downloaded from cBioportal website (https://www.cbioportal.org). The *x*‐axis shows the number of amino acid residues. The red lollipop is the mutation observed in the CRC patient of interest. Type of mutations is shown with lollipop structures, Green = Missense, Gray = Nonsense, Yellow = Splice. ABL1, ABL Proto‐Oncogene 1; ALK, anaplastic lymphoma kinase; FGFR2, Fibroblast Growth Factor Receptor 2; ROS1, ROS proto‐oncogene. Red box highlights the molecular characteristics of analyzed tumor.
**Fig. S4.** Genome alterations. (A) Graphs show fraction genome altered, copy number aberration and copy number heterogeneity. Boxplots indicate the values of CRC cohort patients, and the red triangle refers to our CRC patient of interest. Graphs show mean, max, and min values. (B) Image reported the amplifications and deletions on chromosome arms.
**Fig. S5.** RNA‐Seq analysis of FGFR2 in tumor tissue when compared to normal tissue. RNA‐Seq analysis of FGFR2 in tumor tissue when compared to normal tissue. Boxplots indicate the values of CRC cohort patients, and the red triangle refers to our CRC patient of interest. Graphs show mean, max, and min values.
**Fig. S6.** (A) RNA‐Seq analysis of MLH1 in tumor tissue when compared to normal tissue. Immunohistochemical investigation confirms the lack of MLH1 expression in the analyzed CRC. (B) RNA‐Seq analysis of PMS2 in tumor tissue when compared to normal tissue. Immunohistochemical investigation confirms the high expression of PMS2 in the analyzed CRC. (C) MSH6 in the analyzed colon adenocarcinoma as compared to normal tissue. Immunohistochemical investigation confirms the high expression of MSH6 in the analyzed CRC. (D) Immunohistochemical analysis shows a complete lack of MSH2 expression in the CRC sample; a discrepant finding emerged from the RNA‐Seq data, showing an increase in transcript levels. Scala bars represent 50 μm. Boxplots indicate the values of CRC cohort, and the red triangle refers to our CRC patient of interest.

## Data Availability

The data will be made available upon reasonable request.
