# Supplementary material for: Multi‐omic characterization of consensus molecular subtype 1 (CMS1) colorectal cancer with dampened immune response improves precision medicine
Source: Mol Oncol. 2025 Jul 11;19(12):3486–98. doi: 10.1002/1878-0261.70023 (PMC12688173; doi:10.1002/1878-0261.70023)
Supplement: Supplementary file 1 — Fig. S1. Image shows RNA‐Seq analysis of the JAK/STAT pathway (JAK2, JAK3, and STAT3) in tumor tissue when compared to normal tissue. Boxplots indicate the values of CRC cohort patients, and the red triangle refers to our CRC patient of interest. Graphs show mean, max, and min values. Fig. S2. Immunohistochemical analysis of immune checkpoints and Consensus molecular subtype (CMS) 1 classification. (A) Programmed death‐ligand 1 (PDL‐1) expression in the analyzed colon adenocarcinoma. (B) High‐magnification of panel A shows numerous PDL‐1 positive colon cancer cells. (C) No/moderate expression of Cytotoxic T‐lymphocyte antigen 4 (CTLA4) in colon cancer cells. (D) No expression of Programmed cell death 1 (PD‐1). (E) Classification of CMS1 cases in terms of Microsatellite Instability (MSI) status (blue: MSI‐H), Microsatellite Instability (TMB) (blue: TMB > 75% percentile of all CRC cases), CTLA‐4 and PD‐1 expression (blue: low: tumor/normal log2FC < 1), as well as JAK–STAT and MAPK activity (blue: JAK–STAT tumor/normal differences < 0, MAPK difference > 0). Scale bars represent 50 μm panels A, C, D and 40 μm panel B. Fig. S3. Mutational analysis. (A) The most relevant somatic mutation detected in our colorectal cancer (CRC) patient, along with the currently active clinical trials considering this mutation for targeted therapies. (B–E) Schematic representation of the indicated genes and location of frequent pathogenic mutations described in CRC patients (TCGA, PanCancer Atlas) downloaded from cBioportal website (https://www.cbioportal.org). The x‐axis shows the number of amino acid residues. The red lollipop is the mutation observed in the CRC patient of interest. Type of mutations is shown with lollipop structures, Green = Missense, Gray = Nonsense, Yellow = Splice. ABL1, ABL Proto‐Oncogene 1; ALK, anaplastic lymphoma kinase; FGFR2, Fibroblast Growth Factor Receptor 2; ROS1, ROS proto‐oncogene. Red box highlights the molecular characteristics of analyzed tumor. Fig. S4 [file MOL2-19-3486-s001.zip › Figure legend supplementary figures.pdf]

Supporting Information

Figure S1: JAK/STAT pathway analysis. Image shows RNA Seq analysis of the JAK/STAT pathway (JAK2, JAK3 and STAT3) in tumor tissue when compared to normal tissue. Boxplots indicate the values of CRC cohort patients, and the red triangle refers to our CRC patient of interest. Graphs show mean, max and min values.

Figure S2. Immunohistochemical analysis of immune-checkpoints and Consensus molecular subtype (CMS) 1 classification. A) Programmed death-ligand 1 (PDL-1) expression in the analyzed colon adenocarcinoma. B) High-magnification of panel A shows numerous PDL-1 positive colon cancer cells. C) No/moderate expression of Cytotoxic T-lymphocyte antigen 4 (CTLA4) in colon cancer cells. D) No expression of Programmed cell death 1 (PD-1). E) Classification of CMS1 cases in terms of Microsatellite Instability (MSI) status (blue: MSI-H), Microsatellite Instability (TMB) (blue: TMB > 75% percentile of all CRC cases), CTLA-4 and PD-1 expression (blue: low: tumor/normal log2FC < 1), as well as JAK-STAT and MAPK activity (blue: JAK-STAT tumor/normal differences < 0, MAPK difference > 0). Scale bars represent 50 µm panels A,C,D and 40 µm panel B

Figure S3: Mutational analysis. A) The most relevant somatic mutation detected in our colorectal cancer (CRC) patient, along with the currently active clinical trials considering this mutation for targeted therapies. B-E) Schematic representation of the indicated genes and location of frequent pathogenic mutations described in CRC patients (TCGA, PanCancer Atlas) downloaded from cBioportal website (https://www.cbioportal.org). The x-axis shows the number of amino acid residues. The red lollipop is the mutation observed in the CRC patient of interest. Type of mutations is shown with lollipop structures, Green= Missense, Gray= Nonsense, Yellow= Splice. ALK= anaplastic lymphoma kinase; ABL1= ABL Proto-Oncogene 1; ROS1= ROS proto-oncogene; FGFR2= Fibroblast Growth Factor Receptor 2. Red box highlights the molecular characteristics of analyzed tumor.

Figure S4: Genome alterations. A) Graphs show fraction genome altered, copy number aberration and copy number heterogeneity. Boxplots indicate the values of CRC cohort patients, and the red triangle refers to our CRC patient of interest. Graphs show mean, max and min values. B) Image reported the amplifications and deletions on chromosome arms.

Figure S5: FGFR2 analysis by RNASeq. RNA Seq analysis of FGFR2 in tumor tissue when compared to normal tissue. Boxplots indicate the values of CRC cohort patients, and the red triangle refers to our CRC patient of interest. Graphs show mean, max and min values.

Figure S6 MSI investigations. A) RNA Seq analysis of MLH1 in tumor tissue when compared to normal tissue. Immunohistochemical investigation confirms the lack of MLH1 expression in the analyzed CRC. B) RNA Seq analysis of PMS2 in tumor tissue when compared to normal tissue. Immunohistochemical investigation confirms the high expression of PMS2 in the analyzed CRC. C) MSH6 in the analysed colon adenocarcinoma as compared to normal tissue. Immunohistochemical investigation confirms the high expression of MSH6 in the analyzed CRC. D) Immunohistochemical analysis shows a complete lack of MSH2 expression in the CRC sample; a discrepant finding emerged from the RNASeq data, showing an increase in transcript levels. Scala bars represent 50 µm.
Boxplots indicate the values of CRC cohort, and the red triangle refers to our CRC patient of interest.
