# Supplementary figures and images for: Multi‐omic characterization of consensus molecular subtype 1 (CMS1) colorectal cancer with dampened immune response improves precision medicine
Source: Mol Oncol. 2025 Jul 11;19(12):3486–98. doi: 10.1002/1878-0261.70023 (PMC12688173; doi:10.1002/1878-0261.70023)

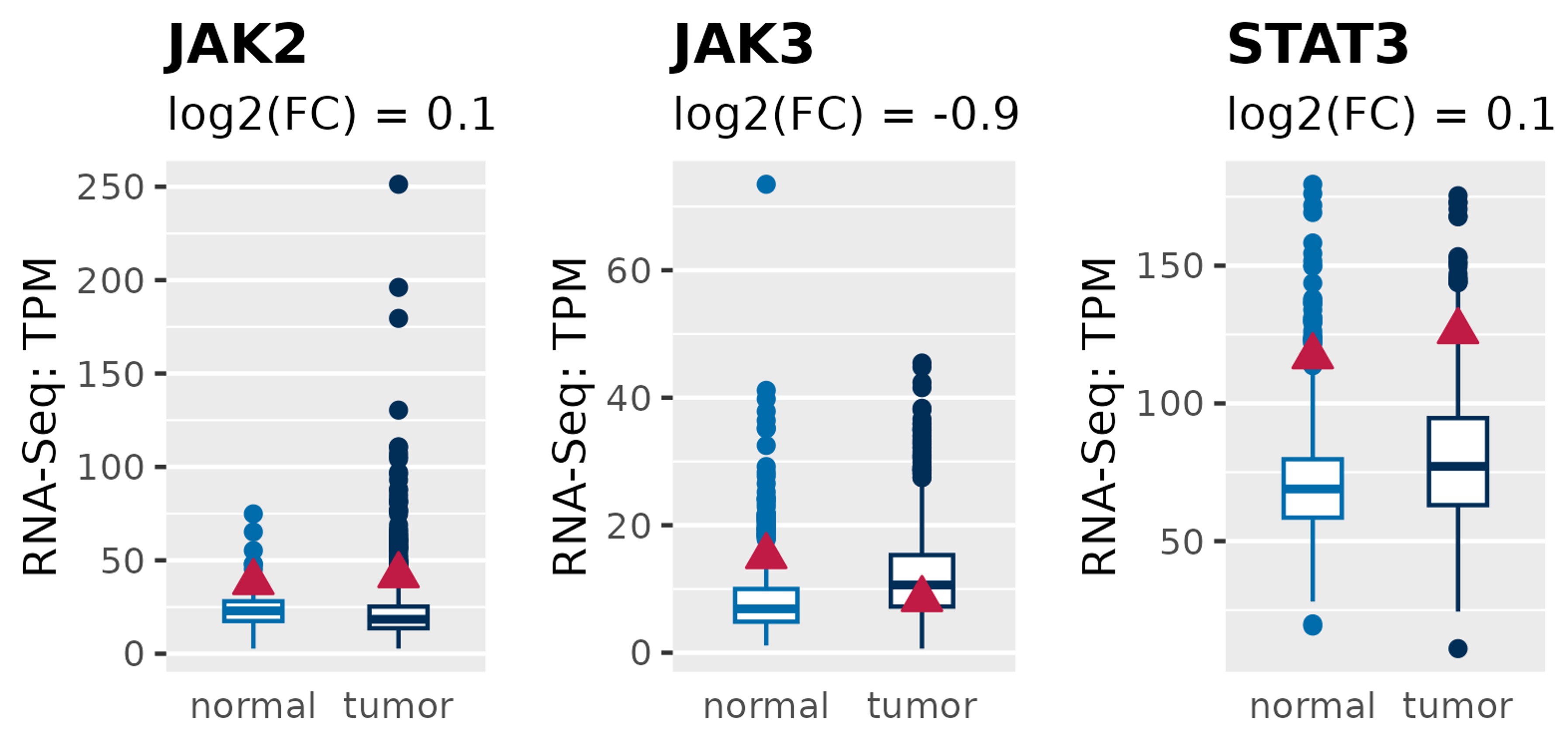

Supplement: Supplementary file 1 — Fig. S1. Image shows RNA‐Seq analysis of the JAK/STAT pathway (JAK2, JAK3, and STAT3) in tumor tissue when compared to normal tissue. Boxplots indicate the values of CRC cohort patients, and the red triangle refers to our CRC patient of interest. Graphs show mean, max, and min values. Fig. S2. Immunohistochemical analysis of immune checkpoints and Consensus molecular subtype (CMS) 1 classification. (A) Programmed death‐ligand 1 (PDL‐1) expression in the analyzed colon adenocarcinoma. (B) High‐magnification of panel A shows numerous PDL‐1 positive colon cancer cells. (C) No/moderate expression of Cytotoxic T‐lymphocyte antigen 4 (CTLA4) in colon cancer cells. (D) No expression of Programmed cell death 1 (PD‐1). (E) Classification of CMS1 cases in terms of Microsatellite Instability (MSI) status (blue: MSI‐H), Microsatellite Instability (TMB) (blue: TMB > 75% percentile of all CRC cases), CTLA‐4 and PD‐1 expression (blue: low: tumor/normal log2FC < 1), as well as JAK–STAT and MAPK activity (blue: JAK–STAT tumor/normal differences < 0, MAPK difference > 0). Scale bars represent 50 μm panels A, C, D and 40 μm panel B. Fig. S3. Mutational analysis. (A) The most relevant somatic mutation detected in our colorectal cancer (CRC) patient, along with the currently active clinical trials considering this mutation for targeted therapies. (B–E) Schematic representation of the indicated genes and location of frequent pathogenic mutations described in CRC patients (TCGA, PanCancer Atlas) downloaded from cBioportal website (https://www.cbioportal.org). The x‐axis shows the number of amino acid residues. The red lollipop is the mutation observed in the CRC patient of interest. Type of mutations is shown with lollipop structures, Green = Missense, Gray = Nonsense, Yellow = Splice. ABL1, ABL Proto‐Oncogene 1; ALK, anaplastic lymphoma kinase; FGFR2, Fibroblast Growth Factor Receptor 2; ROS1, ROS proto‐oncogene. Red box highlights the molecular characteristics of analyzed tumor. Fig. S4 [file MOL2-19-3486-s001.zip › MOL2_70023_f1_S1__.jpg]

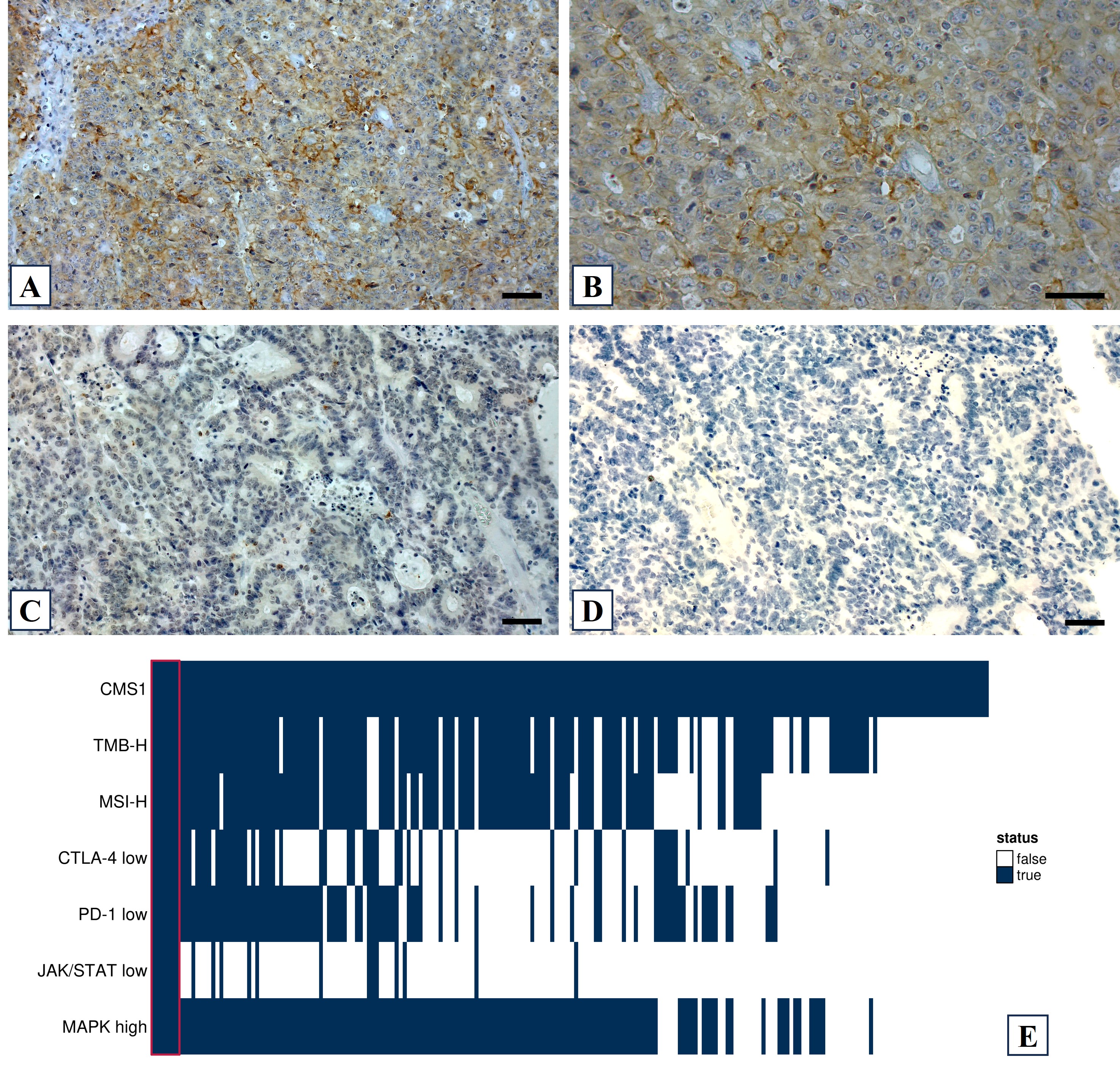

Supplement: Supplementary file 1 — Fig. S1. Image shows RNA‐Seq analysis of the JAK/STAT pathway (JAK2, JAK3, and STAT3) in tumor tissue when compared to normal tissue. Boxplots indicate the values of CRC cohort patients, and the red triangle refers to our CRC patient of interest. Graphs show mean, max, and min values. Fig. S2. Immunohistochemical analysis of immune checkpoints and Consensus molecular subtype (CMS) 1 classification. (A) Programmed death‐ligand 1 (PDL‐1) expression in the analyzed colon adenocarcinoma. (B) High‐magnification of panel A shows numerous PDL‐1 positive colon cancer cells. (C) No/moderate expression of Cytotoxic T‐lymphocyte antigen 4 (CTLA4) in colon cancer cells. (D) No expression of Programmed cell death 1 (PD‐1). (E) Classification of CMS1 cases in terms of Microsatellite Instability (MSI) status (blue: MSI‐H), Microsatellite Instability (TMB) (blue: TMB > 75% percentile of all CRC cases), CTLA‐4 and PD‐1 expression (blue: low: tumor/normal log2FC < 1), as well as JAK–STAT and MAPK activity (blue: JAK–STAT tumor/normal differences < 0, MAPK difference > 0). Scale bars represent 50 μm panels A, C, D and 40 μm panel B. Fig. S3. Mutational analysis. (A) The most relevant somatic mutation detected in our colorectal cancer (CRC) patient, along with the currently active clinical trials considering this mutation for targeted therapies. (B–E) Schematic representation of the indicated genes and location of frequent pathogenic mutations described in CRC patients (TCGA, PanCancer Atlas) downloaded from cBioportal website (https://www.cbioportal.org). The x‐axis shows the number of amino acid residues. The red lollipop is the mutation observed in the CRC patient of interest. Type of mutations is shown with lollipop structures, Green = Missense, Gray = Nonsense, Yellow = Splice. ABL1, ABL Proto‐Oncogene 1; ALK, anaplastic lymphoma kinase; FGFR2, Fibroblast Growth Factor Receptor 2; ROS1, ROS proto‐oncogene. Red box highlights the molecular characteristics of analyzed tumor. Fig. S4 [file MOL2-19-3486-s001.zip › MOL2_70023_f2_S2_.jpg]

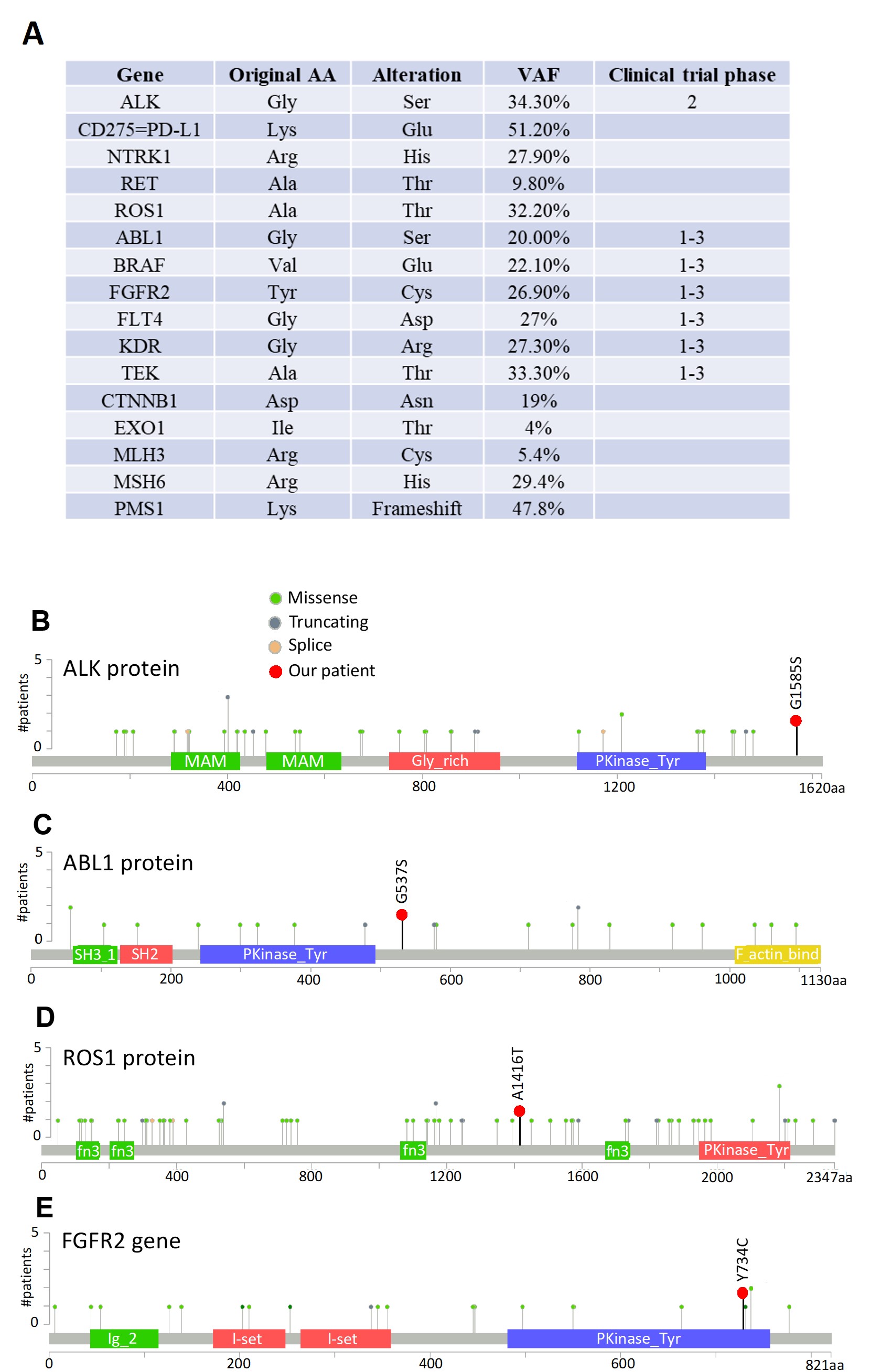

Supplement: Supplementary file 1 — Fig. S1. Image shows RNA‐Seq analysis of the JAK/STAT pathway (JAK2, JAK3, and STAT3) in tumor tissue when compared to normal tissue. Boxplots indicate the values of CRC cohort patients, and the red triangle refers to our CRC patient of interest. Graphs show mean, max, and min values. Fig. S2. Immunohistochemical analysis of immune checkpoints and Consensus molecular subtype (CMS) 1 classification. (A) Programmed death‐ligand 1 (PDL‐1) expression in the analyzed colon adenocarcinoma. (B) High‐magnification of panel A shows numerous PDL‐1 positive colon cancer cells. (C) No/moderate expression of Cytotoxic T‐lymphocyte antigen 4 (CTLA4) in colon cancer cells. (D) No expression of Programmed cell death 1 (PD‐1). (E) Classification of CMS1 cases in terms of Microsatellite Instability (MSI) status (blue: MSI‐H), Microsatellite Instability (TMB) (blue: TMB > 75% percentile of all CRC cases), CTLA‐4 and PD‐1 expression (blue: low: tumor/normal log2FC < 1), as well as JAK–STAT and MAPK activity (blue: JAK–STAT tumor/normal differences < 0, MAPK difference > 0). Scale bars represent 50 μm panels A, C, D and 40 μm panel B. Fig. S3. Mutational analysis. (A) The most relevant somatic mutation detected in our colorectal cancer (CRC) patient, along with the currently active clinical trials considering this mutation for targeted therapies. (B–E) Schematic representation of the indicated genes and location of frequent pathogenic mutations described in CRC patients (TCGA, PanCancer Atlas) downloaded from cBioportal website (https://www.cbioportal.org). The x‐axis shows the number of amino acid residues. The red lollipop is the mutation observed in the CRC patient of interest. Type of mutations is shown with lollipop structures, Green = Missense, Gray = Nonsense, Yellow = Splice. ABL1, ABL Proto‐Oncogene 1; ALK, anaplastic lymphoma kinase; FGFR2, Fibroblast Growth Factor Receptor 2; ROS1, ROS proto‐oncogene. Red box highlights the molecular characteristics of analyzed tumor. Fig. S4 [file MOL2-19-3486-s001.zip › MOL2_70023_f3_S3_.jpg]

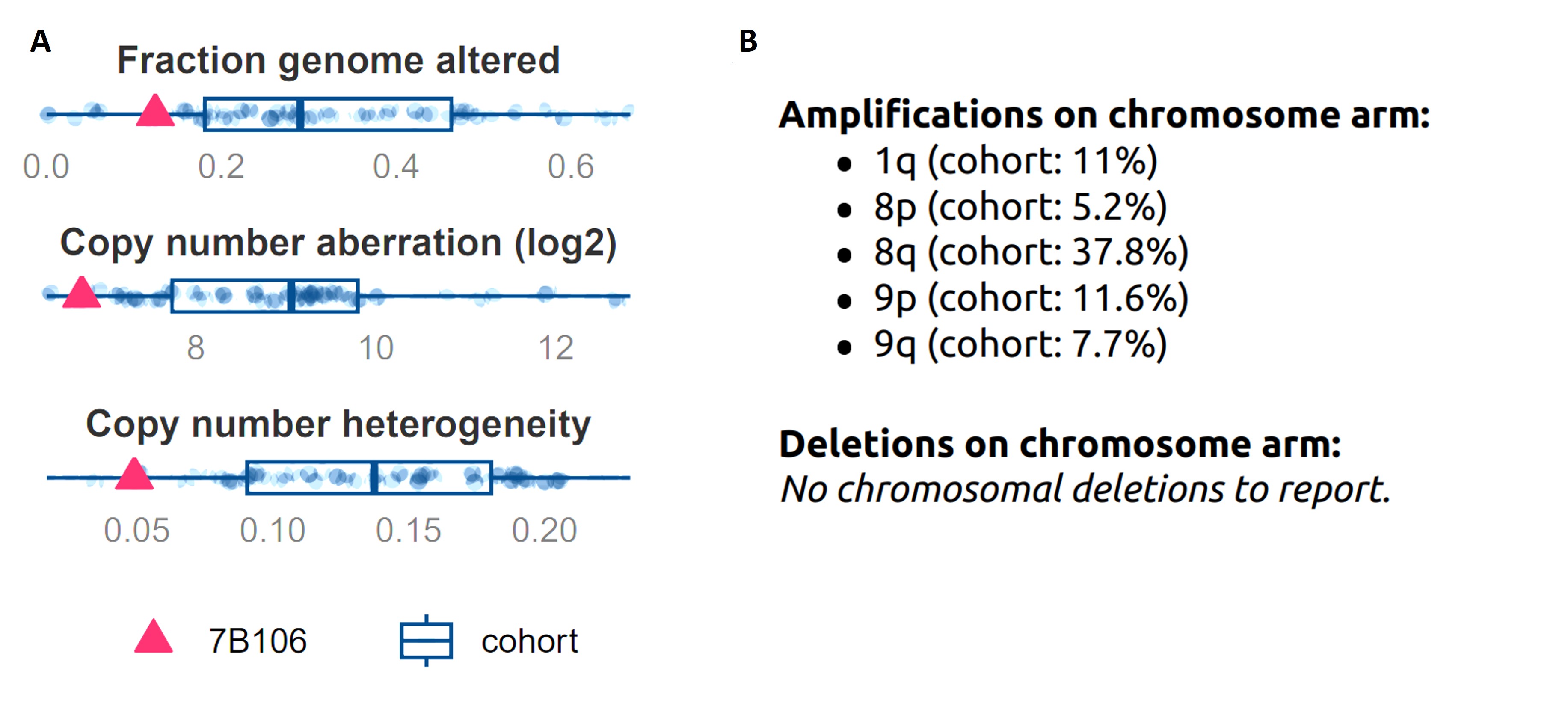

Supplement: Supplementary file 1 — Fig. S1. Image shows RNA‐Seq analysis of the JAK/STAT pathway (JAK2, JAK3, and STAT3) in tumor tissue when compared to normal tissue. Boxplots indicate the values of CRC cohort patients, and the red triangle refers to our CRC patient of interest. Graphs show mean, max, and min values. Fig. S2. Immunohistochemical analysis of immune checkpoints and Consensus molecular subtype (CMS) 1 classification. (A) Programmed death‐ligand 1 (PDL‐1) expression in the analyzed colon adenocarcinoma. (B) High‐magnification of panel A shows numerous PDL‐1 positive colon cancer cells. (C) No/moderate expression of Cytotoxic T‐lymphocyte antigen 4 (CTLA4) in colon cancer cells. (D) No expression of Programmed cell death 1 (PD‐1). (E) Classification of CMS1 cases in terms of Microsatellite Instability (MSI) status (blue: MSI‐H), Microsatellite Instability (TMB) (blue: TMB > 75% percentile of all CRC cases), CTLA‐4 and PD‐1 expression (blue: low: tumor/normal log2FC < 1), as well as JAK–STAT and MAPK activity (blue: JAK–STAT tumor/normal differences < 0, MAPK difference > 0). Scale bars represent 50 μm panels A, C, D and 40 μm panel B. Fig. S3. Mutational analysis. (A) The most relevant somatic mutation detected in our colorectal cancer (CRC) patient, along with the currently active clinical trials considering this mutation for targeted therapies. (B–E) Schematic representation of the indicated genes and location of frequent pathogenic mutations described in CRC patients (TCGA, PanCancer Atlas) downloaded from cBioportal website (https://www.cbioportal.org). The x‐axis shows the number of amino acid residues. The red lollipop is the mutation observed in the CRC patient of interest. Type of mutations is shown with lollipop structures, Green = Missense, Gray = Nonsense, Yellow = Splice. ABL1, ABL Proto‐Oncogene 1; ALK, anaplastic lymphoma kinase; FGFR2, Fibroblast Growth Factor Receptor 2; ROS1, ROS proto‐oncogene. Red box highlights the molecular characteristics of analyzed tumor. Fig. S4 [file MOL2-19-3486-s001.zip › MOL2_70023_f4_s4_.jpg]

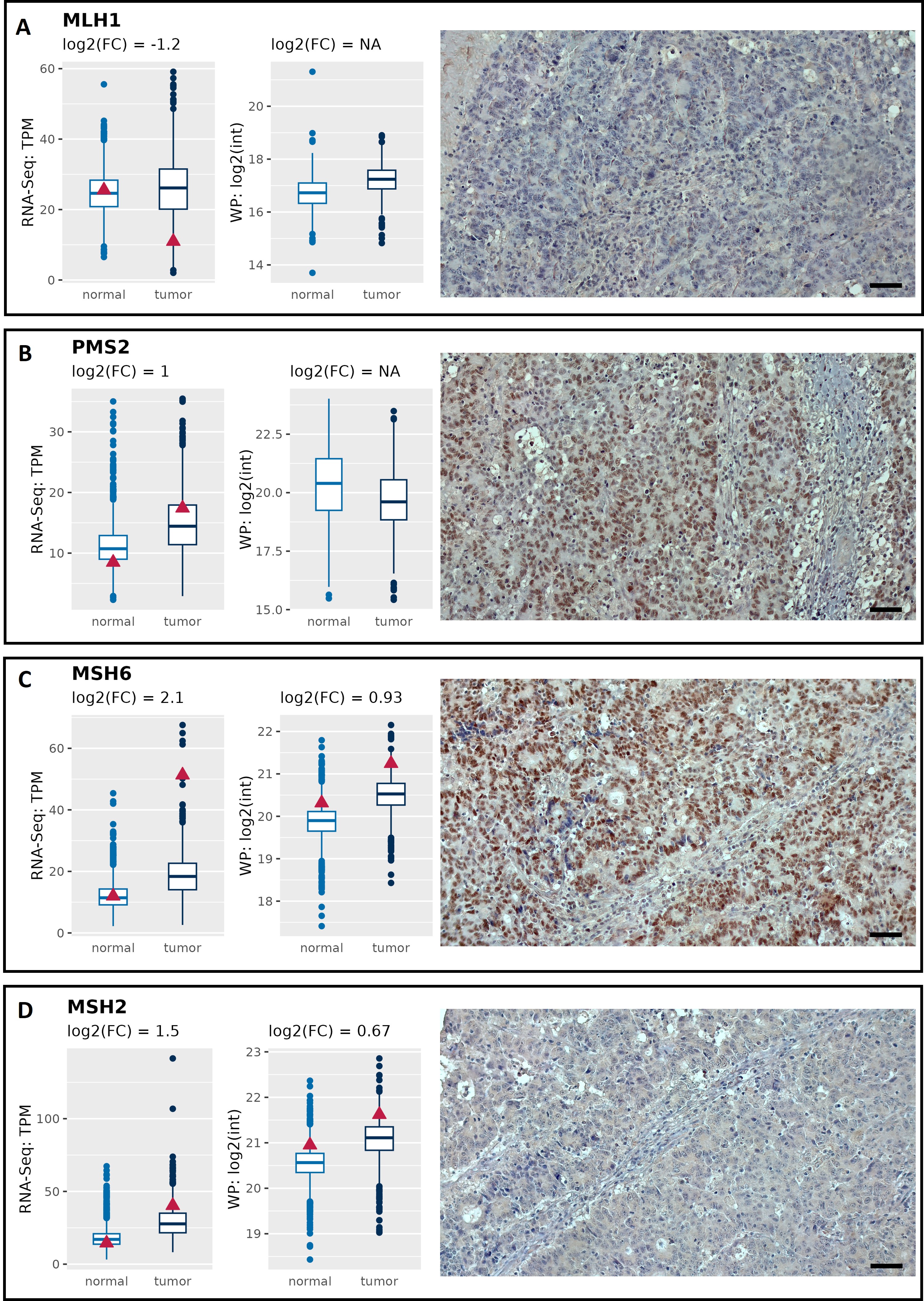

Supplement: Supplementary file 1 — Fig. S1. Image shows RNA‐Seq analysis of the JAK/STAT pathway (JAK2, JAK3, and STAT3) in tumor tissue when compared to normal tissue. Boxplots indicate the values of CRC cohort patients, and the red triangle refers to our CRC patient of interest. Graphs show mean, max, and min values. Fig. S2. Immunohistochemical analysis of immune checkpoints and Consensus molecular subtype (CMS) 1 classification. (A) Programmed death‐ligand 1 (PDL‐1) expression in the analyzed colon adenocarcinoma. (B) High‐magnification of panel A shows numerous PDL‐1 positive colon cancer cells. (C) No/moderate expression of Cytotoxic T‐lymphocyte antigen 4 (CTLA4) in colon cancer cells. (D) No expression of Programmed cell death 1 (PD‐1). (E) Classification of CMS1 cases in terms of Microsatellite Instability (MSI) status (blue: MSI‐H), Microsatellite Instability (TMB) (blue: TMB > 75% percentile of all CRC cases), CTLA‐4 and PD‐1 expression (blue: low: tumor/normal log2FC < 1), as well as JAK–STAT and MAPK activity (blue: JAK–STAT tumor/normal differences < 0, MAPK difference > 0). Scale bars represent 50 μm panels A, C, D and 40 μm panel B. Fig. S3. Mutational analysis. (A) The most relevant somatic mutation detected in our colorectal cancer (CRC) patient, along with the currently active clinical trials considering this mutation for targeted therapies. (B–E) Schematic representation of the indicated genes and location of frequent pathogenic mutations described in CRC patients (TCGA, PanCancer Atlas) downloaded from cBioportal website (https://www.cbioportal.org). The x‐axis shows the number of amino acid residues. The red lollipop is the mutation observed in the CRC patient of interest. Type of mutations is shown with lollipop structures, Green = Missense, Gray = Nonsense, Yellow = Splice. ABL1, ABL Proto‐Oncogene 1; ALK, anaplastic lymphoma kinase; FGFR2, Fibroblast Growth Factor Receptor 2; ROS1, ROS proto‐oncogene. Red box highlights the molecular characteristics of analyzed tumor. Fig. S4 [file MOL2-19-3486-s001.zip › MOL2_70023_f6_S6.jpg]

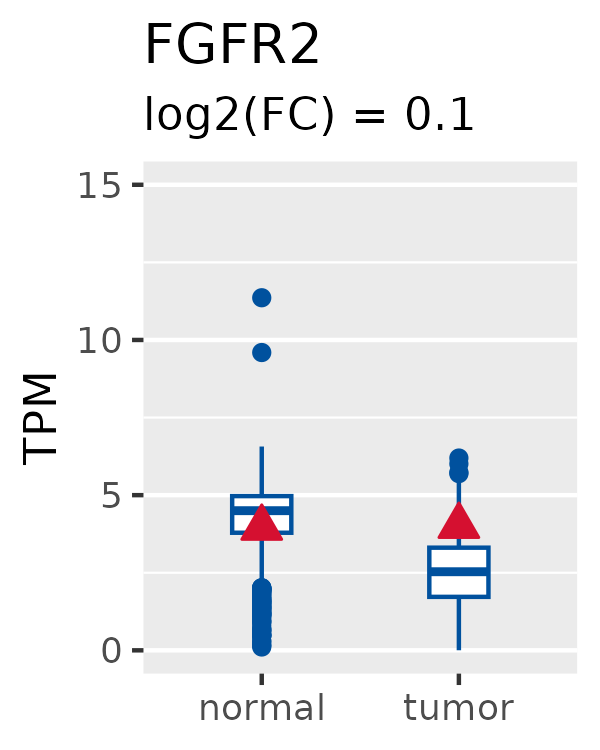

Supplement: Supplementary file 1 — Fig. S1. Image shows RNA‐Seq analysis of the JAK/STAT pathway (JAK2, JAK3, and STAT3) in tumor tissue when compared to normal tissue. Boxplots indicate the values of CRC cohort patients, and the red triangle refers to our CRC patient of interest. Graphs show mean, max, and min values. Fig. S2. Immunohistochemical analysis of immune checkpoints and Consensus molecular subtype (CMS) 1 classification. (A) Programmed death‐ligand 1 (PDL‐1) expression in the analyzed colon adenocarcinoma. (B) High‐magnification of panel A shows numerous PDL‐1 positive colon cancer cells. (C) No/moderate expression of Cytotoxic T‐lymphocyte antigen 4 (CTLA4) in colon cancer cells. (D) No expression of Programmed cell death 1 (PD‐1). (E) Classification of CMS1 cases in terms of Microsatellite Instability (MSI) status (blue: MSI‐H), Microsatellite Instability (TMB) (blue: TMB > 75% percentile of all CRC cases), CTLA‐4 and PD‐1 expression (blue: low: tumor/normal log2FC < 1), as well as JAK–STAT and MAPK activity (blue: JAK–STAT tumor/normal differences < 0, MAPK difference > 0). Scale bars represent 50 μm panels A, C, D and 40 μm panel B. Fig. S3. Mutational analysis. (A) The most relevant somatic mutation detected in our colorectal cancer (CRC) patient, along with the currently active clinical trials considering this mutation for targeted therapies. (B–E) Schematic representation of the indicated genes and location of frequent pathogenic mutations described in CRC patients (TCGA, PanCancer Atlas) downloaded from cBioportal website (https://www.cbioportal.org). The x‐axis shows the number of amino acid residues. The red lollipop is the mutation observed in the CRC patient of interest. Type of mutations is shown with lollipop structures, Green = Missense, Gray = Nonsense, Yellow = Splice. ABL1, ABL Proto‐Oncogene 1; ALK, anaplastic lymphoma kinase; FGFR2, Fibroblast Growth Factor Receptor 2; ROS1, ROS proto‐oncogene. Red box highlights the molecular characteristics of analyzed tumor. Fig. S4 [file MOL2-19-3486-s001.zip › MOL2_70023_f7_7B106_FGFR2_S5.png]
